# Pulmonary Tuberculosis With Left Pleural Effusion and Lichen Scrofulosorum: A First-of-Its-Type Report

**DOI:** 10.7759/cureus.57968

**Published:** 2024-04-10

**Authors:** Sankalp Yadav

**Affiliations:** 1 Medicine, Shri Madan Lal Khurana Chest Clinic, New Delhi, IND

**Keywords:** pleural effusion, pulmonary tuberculosis, tuberculids, mycobacterium tuberculosis, lichen scrofulosorum, cutaneous tuberculosis

## Abstract

Tuberculosis is prevalent in high-burden countries, but its cutaneous form, tuberculid, is rare and often misdiagnosed. Lichen scrofulosorum, a type of tuberculid, is uncommon and typically affects children and young adults, sometimes alongside other tuberculosis symptoms. Herein, a very rare case of lichen scrofulosorum in a 20-year-old Indian male with an underlying focus of tuberculosis in the lungs and pleura is presented. Prompt treatment after detailed lab work backed by clinical assessment helped in establishing the diagnosis. He was put on antitubercular therapy, which led to a marked improvement in skin and pulmonary lesions. However, he was lost to follow-up.

## Introduction

Extrapulmonary tuberculosis, although less common than pulmonary tuberculosis, is documented in the published literature, constituting approximately 8.4-13.7% of all tuberculosis cases [1]. It can manifest in various forms, such as cutaneous, pleural, abdominal, bone and joint, hepatic, splenic, lymphatic, and meningeal [2]. Cutaneous tuberculosis, in particular, is rare, with approximately 5.9 cases per 1,000 individuals and a prevalence of 0.25-0.6%, according to Indian studies. It is caused by *Mycobacterium bovis*, *Mycobacterium tuberculosis* complex, and occasionally, Bacillus Calmette-Guérin (BCG) vaccination [3].

A manifestation of cutaneous tuberculosis, tuberculids are caused by delayed hypersensitivity reactions in people with strong cell-mediated immunity to *M. tuberculosis* or mycobacterial antigens [4]. Around the world, the prevalence of tuberculids among cases of cutaneous tuberculosis ranges from 4% to 44.2%. In many regions of the world, erythema induratum of Bazin is thought to be the most prevalent tuberculid, yet Indian studies indicate that lichen scrofulosorum is the most common [5].

A rare case of a young Indian male is presented here. The case is remarkable as there were pulmonary foci and extrapulmonary tuberculosis without a history of disease in a cutaneous tuberculosis presentation.

## Case presentation

A 20-year-old nondiabetic Indian male of a low socioeconomic background presented with complaints of fever without chills off and on for four weeks, left-sided chest pain for two weeks, and multiple skin lesions over his trunk for two weeks. He had been consulting local clinicians, who prescribed him anti-allergics (details not known) and paracetamol. His fever subsided after this treatment, but the skin lesions continued to cover his trunk. He was a student with no history of substance abuse, skin infections, allergies, or any major medical (including tuberculosis) or surgical intervention in the past. There was no history of weight loss, cough, loss of appetite, or night sweats.

**Figure 1:**
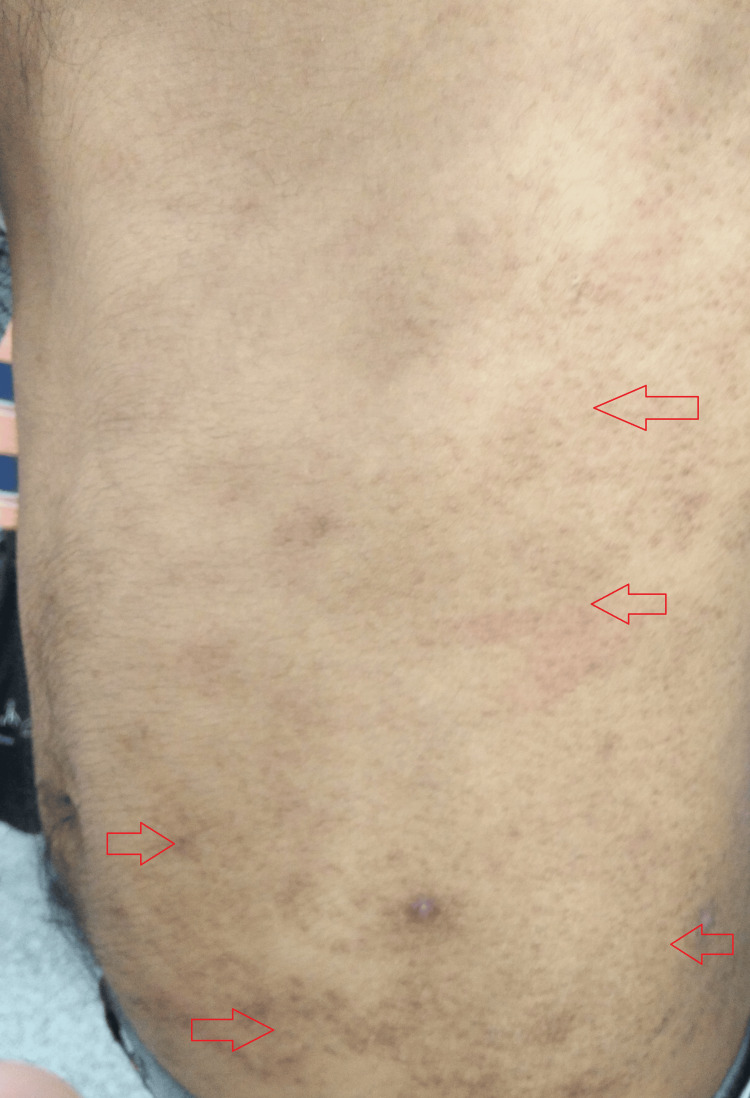
Gross image at presentation showing multiple, asymptomatic, flat-topped, follicular and perifollicular, skin-colored, lichenoid, mostly grouped papules over the patient’s trunk

**Figure 2:**
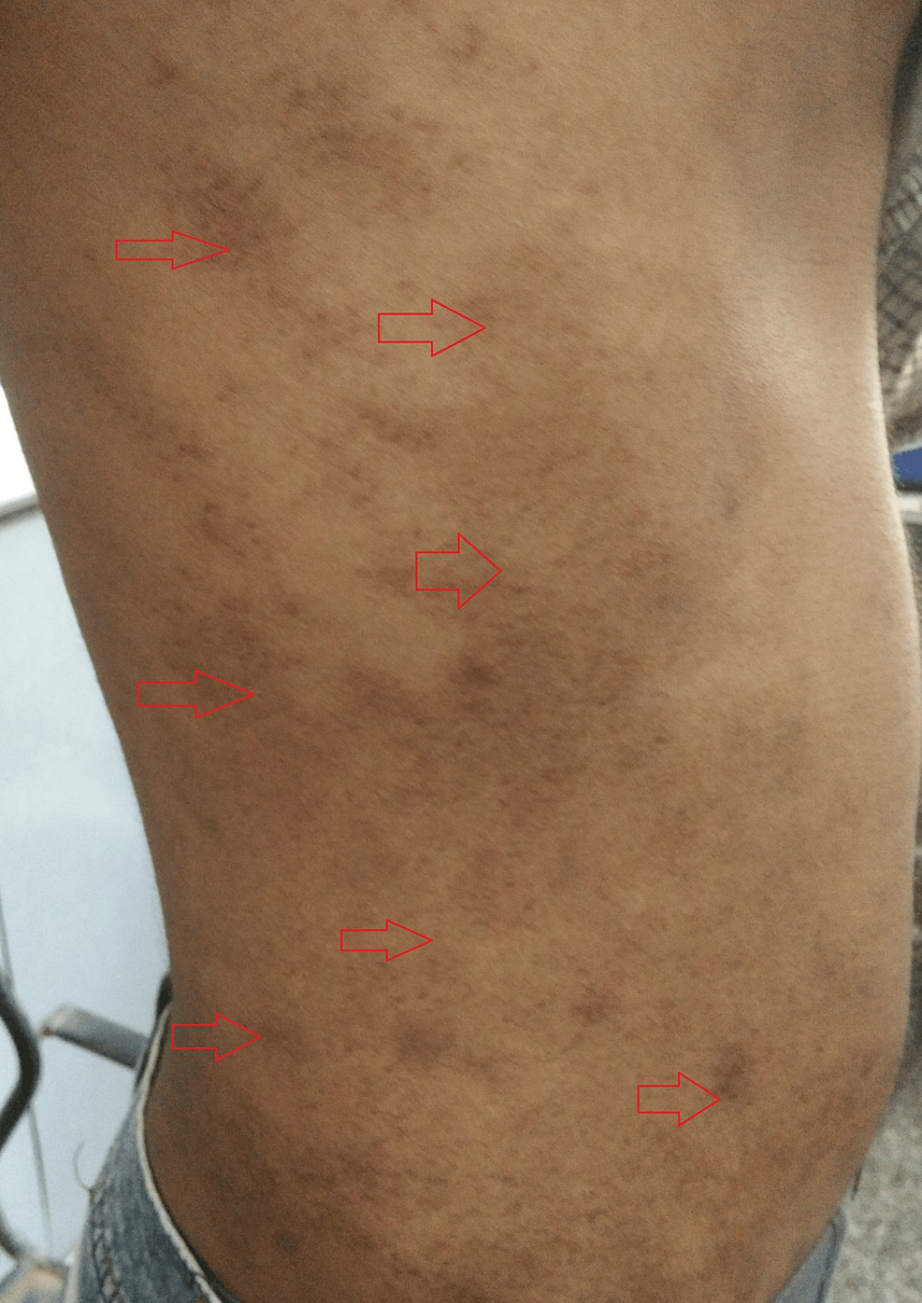
Gross image at presentation showing multiple, asymptomatic, flat-topped, follicular and perifollicular, skin-colored, lichenoid, mostly grouped papules over the patient’s trunk

The general examination was suggestive of a hemodynamically stable man with an ectomorphic build. Systemic examination was remarkable for reduced breath sounds and decreased tactile and vocal fremitus over the left lower lobe of the lung. Local examination of the skin was suggestive of multiple, asymptomatic, flat-topped, follicular and perifollicular, skin-colored, lichenoid, mostly grouped papules over his trunk (Figures 1, 2).

**Figure 3:**
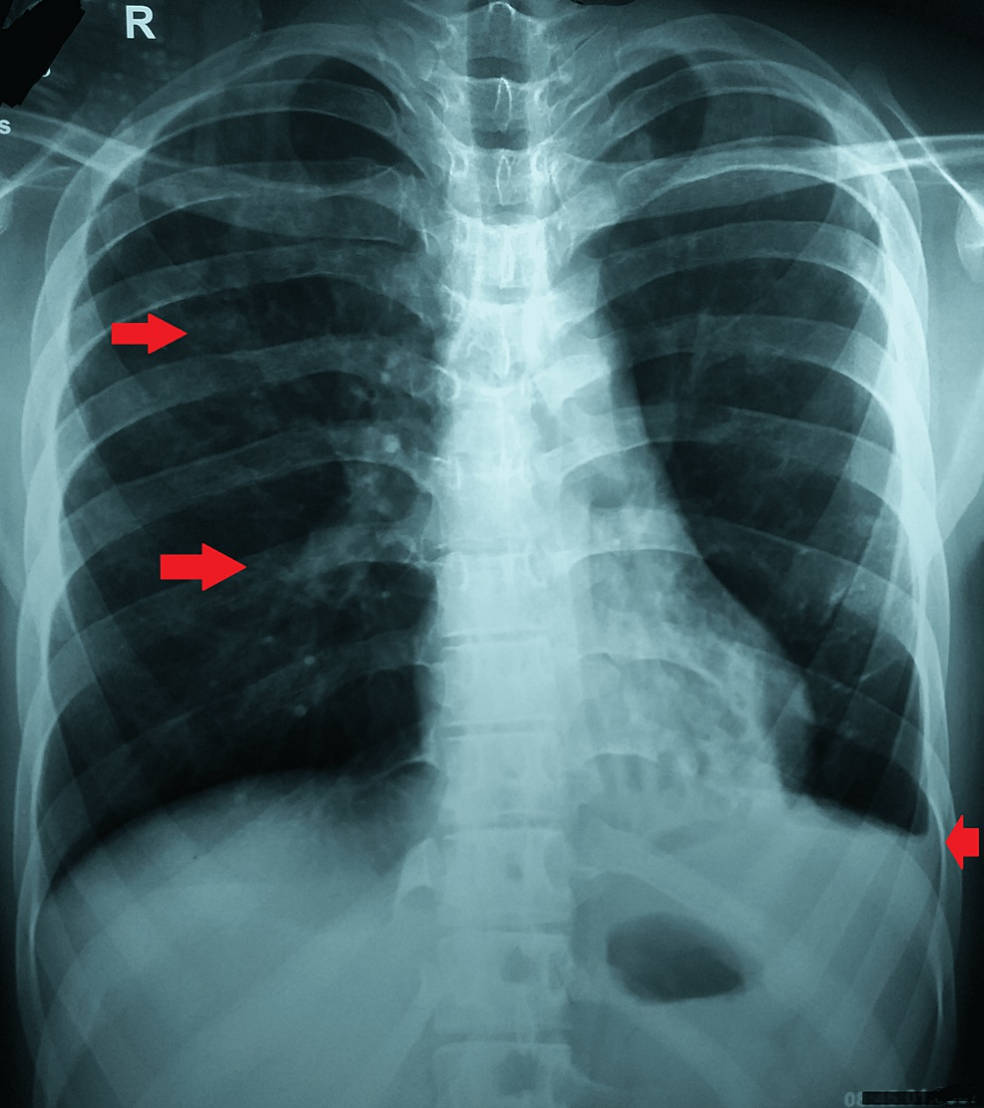
Chest radiograph showing pulmonary opacities and pleural effusion

The patient underwent blood investigations and chest radiographs. The lab panel was remarkable for a raised, i.e., 85 mm/hour, erythrocyte sedimentation rate. His HIV (I and II) were nonreactive, but the Mantoux test was positive, with an induration of 10 × 10 mm. Dermoscopy showed pale, round, monomorphic, grouped perifollicular dots with a central brown follicular plug and marginal hyperpigmentation. A chest radiograph was suggestive of patchy air opacification of the right upper and middle lobes with a left-sided blunting of the costophrenic angle (Figure 3).

An ultrasound of the chest confirmed a left-sided mild pleural effusion (about 40 ml). However, an ultrasound-guided diagnostic pleural tap gave a dry tap. Moreover, induced sputum microscopy for acid-fast bacilli and cartridge-based nucleic acid amplification of the induced sputum were negative. A punch biopsy under local anesthesia was done at a different center, and the samples were sent for histopathology. It showed focal lichenoid tuberculoid noncaseating granulomatous inflammation in the upper dermis. The granuloma was centered around a hair follicle and was also seen in the overlying papillary dermis, forming a longitudinal perifollicular granuloma. It was made of abundant epithelioid cells, histiocytes, and a few Langhans giant cells surrounded by a narrow rim of lymphocytes. The overlying epidermis showed compact orthohyperkeratosis and acanthosis, with focal atrophy at places of granuloma. The reports were suggestive of lichen scrofulosorum. Special staining, tissue culture on a Mycobacteria growth indicator tube, and polymerase chain reaction from the biopsy samples were all negative for *M. tuberculosis*.

A definitive diagnosis of pulmonary tuberculosis with left pleural effusion and lichen scrofulosorum was made on the basis of histological findings, radiographic investigations, a positive Mantoux test, and clinical presentation. His antituberculous treatment was initiated per the national guidelines with fixed-dose combinations of four drugs (rifampicin, pyrazinamide, ethambutol, and isoniazid in the intensive phase) followed by three drugs (rifampicin, ethambutol, and isoniazid in the continuation phase) for the remaining duration of treatment. There was a remarkable improvement in his symptoms over the next two months, along with the clearing of skin lesions. He was regularly followed up for three months but was lost afterward due to unknown reasons.

## Discussion

In 1896, Darier introduced the idea of tuberculid. The characteristics of the tuberculids were explained by a hypersensitivity reaction to mycobacteria or their parts discharged from a different site of apparent or past tuberculous infection, in contrast to “true” cutaneous tuberculosis. A substantially positive tuberculin skin test, evidence of concurrent or prior tuberculosis, and a fast response to antituberculous chemotherapy are important indicators of tuberculid disease. They fall into one of three categories: first, lichen scrofulosorum; second, Bazin’s erythema induratum; and third, papulonecrotic tuberculid [6].

Lichen scrofulosorum, also known as “tuberculosis cutis lichenoides,” is classified as a true tuberculid and is a rare clinicopathologic manifestation of tuberculids [5,7]. It was first detailed by Hebra in 1860 [6]. It is not the outcome of a local cutaneous tuberculosis infection; rather, it is a genuine hypersensitivity reaction. Rarely, lichen scrofulosorum occurs following the BCG immunization and is typically observed in children and young adults with systemic tuberculosis, either pulmonary or extrapulmonary [8].

Lichen scrofulosorum is frequently found in children; however, in the present case, it was an adult. A study of 221 patients revealed that 70.5% of them were children. The trunk was the primary area affected (98.6%), followed by the lower limb (25.33%), upper limb (15.83%), face (5%), and external genitalia (3.6%) [5,9]. In the present case, the trunk was also mainly involved.

Clinically, it is defined by small, smooth-surfaced, skin-colored perifollicular papules that are grouped together; on rare occasions, spiny projections with fine scales may be visible. The lesions seem to have noncaseating epithelioid cell granulomas around dermal appendages and the outer dermis. It is rare to find tubercle bacilli in histology specimens, and they are not able to be cultured [6]. The same was seen in the present case.

Histopathology, tuberculin skin tests, and indications of systemic tuberculosis are the main approaches used to diagnose lichen scrofulosorum. Diagnosis is aided by strong Mantoux positives, distinctive histological characteristics, and the lack of *M. tuberculosis* in culture. Treatment is conservative with antituberculous drugs as used for systemic tuberculosis [5,6]. All these were noted in the present case.

A detailed literature review revealed that a case of pulmonary tuberculosis with concurrent left pleural effusion and lichen scrofulosorum has never been reported. In a series of eight cases by Beena et al., five had pulmonary tuberculosis, while one had pleural effusion with concomitant lichen scrofulosorum [10]. However, none of these had all three conditions, i.e., pulmonary tuberculosis, left pleural effusion, and lichen scrofulosorum, together. Moreover, their case of pleural effusion had a history of tuberculosis, which was not reported in the present case.

Lichen scrofulosorum is often overlooked due to the morphological resemblances between lichen scrofulosorum and other closely related illnesses, as well as the condition’s rising and declining course. A strong index of suspicion and an adequate evaluation of the underlying tuberculosis focus are required for a successful diagnosis [7,11].

## Conclusions

A first-of-its-type case of concurrent left pleural effusion and lichen scrofulosorum in an Indian immunocompetent male is presented. The case sheds light on the importance of documenting such rare presentations, as even in endemic countries, these cases are hardly reported. Additionally, this case will help in creating awareness about such rare presentations of a very common disease, thereby helping the clinicians to timely treat these cases to prevent exacerbations of the infections.
